# Effects of Post-Resuscitation Treatment with N-acetylcysteine on Cardiac Recovery in Hypoxic Newborn Piglets

**DOI:** 10.1371/journal.pone.0015322

**Published:** 2010-12-21

**Authors:** Jiang-Qin Liu, Tze-Fun Lee, David L. Bigam, Po-Yin Cheung

**Affiliations:** 1 Department of Pediatrics, University of Alberta, Edmonton, Alberta, Canada; 2 Department of Surgery, University of Alberta, Edmonton, Alberta, Canada; University of Giessen Lung Center, Germany

## Abstract

**Aims:**

Although N-acetylcysteine (NAC) can decrease reactive oxygen species and improve myocardial recovery after ischemia/hypoxia in various acute animal models, little is known regarding its long-term effect in neonatal subjects. We investigated whether NAC provides prolonged protective effect on hemodynamics and oxidative stress using a surviving swine model of neonatal asphyxia.

**Methods and Results:**

Newborn piglets were anesthetized and acutely instrumented for measurement of systemic hemodynamics and oxygen transport. Animals were block-randomized into a sham-operated group (without hypoxia-reoxygenation [H–R, n = 6]) and two H-R groups (2 h normocapnic alveolar hypoxia followed by 48 h reoxygenation, n = 8/group). All piglets were acidotic and in cardiogenic shock after hypoxia. At 5 min after reoxygenation, piglets were given either saline or NAC (intravenous 150 mg/kg bolus + 20 mg/kg/h infusion) via for 24 h in a blinded, randomized fashion. Both cardiac index and stroke volume of H-R controls remained lower than the pre-hypoxic values throughout recovery. Treating the piglets with NAC significantly improved cardiac index, stroke volume and systemic oxygen delivery to levels not different from those of sham-operated piglets. Accompanied with the hemodynamic improvement, NAC-treated piglets had significantly lower plasma cardiac troponin-I, myocardial lipid hydroperoxides, activated caspase-3 and lactate levels (vs. H-R controls). The change in cardiac index after H-R correlated with myocardial lipid hydroperoxides, caspase-3 and lactate levels (all p<0.05).

**Conclusions:**

Post-resuscitation administration of NAC reduces myocardial oxidative stress and caused a prolonged improvement in cardiac function and in newborn piglets with H-R insults.

## Introduction

There are reports showing cardiovascular dysfunction occurs in 29–67% of asphyxiated neonates using different diagnostic criteria and methods [1], [2], [3]. The outcome of asphyxiated neonates with severe cardiovascular dysfunction is poor and more cardiac support was required during the recovery in these cases [4], [5]. The overproduction of reactive oxygen and nitrogen species during the reperfusion/reoxygenation after hypoxic-ischemic insult will lead to a second strike to myocardial tissues [6], [7]. It is therefore expected that treating the patients suffering from hypoxic-reoxygenation (H–R)/ischemic-reperfusion (I–R) events with antioxidants would minimize the cardiac injury induced by reactive oxygen species (ROS) through various mechanisms [8], [9], [10]. Indeed, the cardiac protective effects of antioxidants have been confirmed in numerous studies of hypoxia-reoxygenation injury [11], [12]. N-Acetylcysteine has been shown to protect various organs against injury after I–R or H–R [13], [14]. Other than acting as a ROS scavenger, NAC is a precursor of L-cysteine and reduced glutathione [15]. It releases thione and converts glutathione into reduced form of GSH which is exhausted during hypoxia and ischemia [16]. Moreover, NAC has been shown to prevent H–R or I–R induced injury via both apoptotic and inflammatory pathways which include the inhibition of NF-kappa B expression as well as caspase-3 activity [17], [18].

Previously in an acute piglet model of neonatal asphyxia, we showed that intravenous infusion of NAC improved cardiac output, stroke volume and systemic oxygen delivery without any changes in mean arterial pressure (MAP) and heart rate [9]. Its beneficial effects might be related to the prompt replenishment of reduced glutathione, scavenging tissue hydrogen peroxide [19] and decreasing lipid hydroperoxides [20]. However, the cardioprotective effect of NAC needs to be further studied at a later stage after resuscitation since the asphyxiating event also has prolonged effects on cardiac function [21]. Abnormal electrocardiography, poor left ventricular function, elevated plasma concentrations of creatinine kinase and cardiac troponins have been observed in asphyxiated neonates at 24–72 h after birth [21], [22], [23]. Similarly, plasma troponin I of neonates with cardiac dysfunction remains elevated at more than 72 h after birth [24]. Taken together, these results indicate that cardiac dysfunction of asphyxiated neonates persists more than 24 h after I–R or H–R insults. Although NAC has been shown to have prolonged cardiac protective effect in various adult animal models [25], [26], limited studies have been carried out to examine its prolonged effect in neonates whose anti-oxidant system is compromised especially with asphyxia.

Using a surviving swine model of neonatal asphyxia, we investigated the effects of NAC on cardiac function as well as its underlying mechanisms after H–R. We hypothesized that the post-resuscitation administration of NAC in asphyxiated newborn piglets would improve the systemic haemodynamics and oxygen transport with the attenuation of oxidative stress in the myocardium.

## Methods

All experiments were conducted in accordance with the guidelines of Canadian Council of Animal Care (2001) and approved by the Animal Care and Use Committee: Health Sciences, University of Alberta (ACUC: HS Protocol #238/06/10D). Male newborn Yorkshire-Landrace piglets 1 day of age weighing 1.6 to 2.5 kg (mean body weight  =  1.93±0.04 kg) were used.

### Anaesthesia

The animal preparation was similar to that described previously [9]. Briefly, anesthesia was initially maintained with inhaled isoflurane (2–3%), which was then switched with fentanyl (0.005–0.05 mg/kg/h), midazolam (0.1–0.2 mg/kg/h) and pancuronium (0.05–0.1 mg/kg/h) once mechanical ventilation was commenced. Oxygen saturation was continuously monitored with a pulse oximeter (Nellcor, Hayward, CA), and heart rate and blood pressure were measured with a 78833B monitor (Hewlett Packard Co., Palo Alto, CA). Fractional inspired oxygen concentration (FiO_2_) was measured by an oxygen monitor (Ohmeda Medical, Laurel, MD) and maintained at 0.21–0.24 for oxygen saturation between 90 and 97%. Maintenance fluids during experimentation consisted of 5% dextrose at 10 ml/kg/h and 0.9% normal saline solution at 2 ml/kg/h. The body temperature was maintained at 38.5–39.5°C using an overhead warmer and a heating pad.

### Surgical preparation

Argyle catheters (5F; Sherwood Medical Co., St. Louis, MO) were inserted via the right femoral artery and vein for continuous measurement of MAP and central venous pressure, respectively. All medications and fluids were administered via the femoral venous catheter. Via a tracheotomy, pressure-controlled assisted ventilation was commenced (Model IV-100, Sechrist Industries Inc., Anaheim, CA) with pressure of 20/4 cm H_2_O at a rate of 18–20 breaths/min. A left anterior thoracotomy was performed to expose the main pulmonary artery. A 20G Insyte-W™ catheter (Becton Dickinson Infusion Therapy Systems Inc., Sandy, UT) was inserted into the root of the main pulmonary artery for the measurement of mean pulmonary artery pressure (PAP) and mixed venous blood oxygen saturation. A 6-mm transit time ultrasound flow probe (6SB, Transonic Systems Inc., Ithaca, NY) was placed around the main pulmonary artery to measure the blood flow as a surrogate of cardiac output.

### Experimental protocol (Fig. 1)

**Figure 1:**
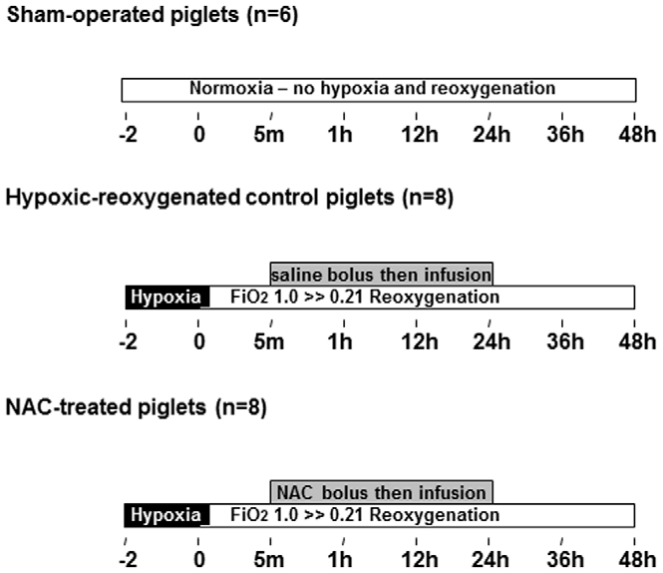
Experimental protocol.

After surgery, animals were stabilized for at least 60 min. Piglets were block-randomized into a sham-operated group (without H–R, but was ventilated with FiO_2_ of 0.21 throughout the experimental period, n = 6) or two H–R experimental groups (n = 8 each) with 2 h hypoxia induced by decreasing the FiO_2_ to 0.10–0.13 using nitrogen and oxygen gas mixture to achieve severe hypoxemia (partial pressure of oxygen [PaO_2_] 20–40 mmHg) for 2 h. After hypoxia, piglets were resuscitated with a FiO_2_ of 1.0 for 1 h, followed by 0.21 for the reminder of the experimental period. Five minutes after reoxygenation, piglets were received either saline (H–R control, 3 ml/kg bolus + 2 ml/kg/h infusion, i.v.) or NAC (150 mg/kg bolus + 20 mg/kg/h, i.v) for 24 h in a blinded, randomized fashion. The dosage and regimen of NAC was based on the clinical protocol used for acetaminophen overdose and our previous studies [9], [19]. During the 48 h of observation, the piglet was placed under a radiator and continuously cared by two experienced team members (JQL and TFL) alternatively who did not know the randomization. Peak inspiratory pressure (18–25 cm H_2_O) and respiratory rate (12–20 breaths/min) were adjusted to maintain normocapnia. The dosages of fentanyl, midazolam and pancuronium were adjusted to maintain minimum body movements throughout the experimental period. Propofol (0.1–0.2 mg/kg/h) was given as needed to maintain anesthesia. A gastric tube was inserted into the stomach orally and drained if needed. A 20G Insyte-W angiocatheter was inserted into bladder transcutaneously to drain the urine. Blood gases were studied every 30 min during hypoxia and 100% oxygen of reoxygenation, every 4 h within first 24 h and every 6 h within second 24 h. At the end of the experiment, the piglet was euthanized with an overdose of pentobarbital (100 mg/kg, i.v.). Left ventricle was removed rapidly and flash-frozen in liquid nitrogen and stored at -80°C for subsequent analysis.

### Hemodynamic recordings and calculations

Hemodynamic parameters (heart rate, MAP, central venous pressure and pulmonary artery flow) were recorded at specific predetermined time points at baseline throughout hypoxia and reoxygenation. Cardiac index (CI), a surrogate estimated by the pulmonary artery flow because of the existence of patent ductus arteriosus, was corrected for individual piglet mass. Stroke volume index, oxygen content, systemic oxygen delivery, consumption and oxygen extraction ratio were calculated using standard formula as below: 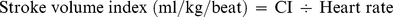


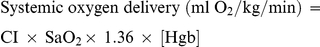


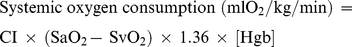


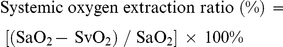



### Biochemical analysis

Myocardial tissues were homogenized with 10 µl/mg of 50 mM phosphate buffer containing 1 mM EDTA (pH 7.0). The tissue levels of oxidized and total glutathione (GSSG and GSH, respectively), lipid hydroperoxides (LPO) and activated caspase-3 were measured using commercially available assay kits (#703002, #705002, #10009135, respectively, Cayman Chemical, Ann Arbor, MI). Tissue lactate was assayed by enzymatic spectrometric methods. Plasma cTnI concentration was measured using a commercially available ELISA kit (#2010-4-HS, Life Diagnostics, West Chester, PA). The protein content was determined by bicinchoninic acid assay kit (Sigma-Aldrich Canada Ltd., Oakville, ON).

### Statistical analysis

All results were expressed as mean±SEM. One- and two-way analysis of variance (ANOVA) tests were used to study the differences among groups as appropriate. Post-hoc testing with Tukey Method was performed for pairwise comparisons with the H–R control group. Non-parametric parameters of cardiac Troponin I were analyzed using ANOVA on rank and Dunn's tests for multiple comparisons. Correlation between variables was studied by Pearson Moment test. Statistical analyses were performed using SigmaStat® (V. 2.0, Jandel Co.). Significance was set at p<0.05.

## Results

There were no differences in body weight, age, hemodynamic and blood gases parameters at baseline among experimental groups.

### Effect of NAC treatment on arterial blood gas and acid-base status

Metabolic acidosis developed after 2 h of normocapnic alveolar hypoxia (Table 1). Upon reoxygenation, PaO_2_, pH and base excess levels of piglets recovered gradually towards the respective normoxic baseline values and were not significantly different from those of sham-operated piglets at the end of experiment. Treating the animals with NAC did not cause any significant changes in blood gas and acid-base status during reoxygenation recovery (Table 1).

**Table 1:** Changes in arterial blood gas during hypoxia and reoxygenation.

	Baseline	End of hypoxia	Reoxygenation (hours)					
			1	4	8	16	24	48
**pH**								
Sham	7.47±0.02	7.45±0.02*	7.47±0.02*	7.45±0.02	7.46±0.01	7.45±0.01	7.44±0.02	7.45±0.02
Control	7.47±0.02	7.05±0.02	7.33±0.02	7.45±0.02	7.45±0.02	7.39±0.02	7.37±0.02	7.39±0.04
NAC	7.48±0.02	7.04±0.02	7.33±0.03	7.46±0.02	7.49±0.02	7.46±0.02	7.41±0.01	7.47±0.01
**PaCO_2_ (mmHg)**								
Sham	35±2	39±1	38±1	39±1	38±1	37±2	38±2	38±1
Control	35±2	42±3	36±2	37±1	36±2	44±2	45±2	43±2
NAC	34±1	41±2	36±1	37±2	37±1	39±2	41±2	37±1
**PaO_2_ (mmHg)**								
Sham	65±2	62±1*	60±2*	60±2	62±3	59±2	62±2	64±2
Control	64±2	30±2	346±26	65±3	59±3	62±3	62±3	59±7
NAC	70±2	35±5	367±21	59±1	57±2	60±2	62±3	61±3
**Arterial O_2_ saturation (%)**								
Sham	95±1.0	94±0.6*	93±0.8*	93±0.9	93±0.5	93±0.7	93±0.8	93±1.4
Control	95±1.0	31±1.4	100±0.3	94±0.7	92±1.3	92±1.0	92±1.4	91±1.6
NAC	95±0.6	30±3.1	100±0.2	92±0.6	94±1.5	93±1.0	93±1.4	94±1.0
**Base Excess (mmol/L)**								
Sham	2.6±1.3	3.3±1.3*	3.2±1.2*	2.7±1.0	2.7±1.0	2.9±0.7	3.0±0.8	2.6±0.6
Control	2.5±0.7	−18.4±0.7	−6.3±0.7	2.1±0.9	2.8±0.5	2.1±0.7	2.2±0.8	2.1±0.7
NAC	2.6±0.8	−18.6±0.9	−5.0±1.2	3.1±0.8	4.3±0.7	3.3±0.8	2.8±0.6	2.6±1.1

*Significantly different from the H−R control group (p<0.05).

### Effects of NAC treatment on systemic hemodynamic parameters

**Figure 2:**
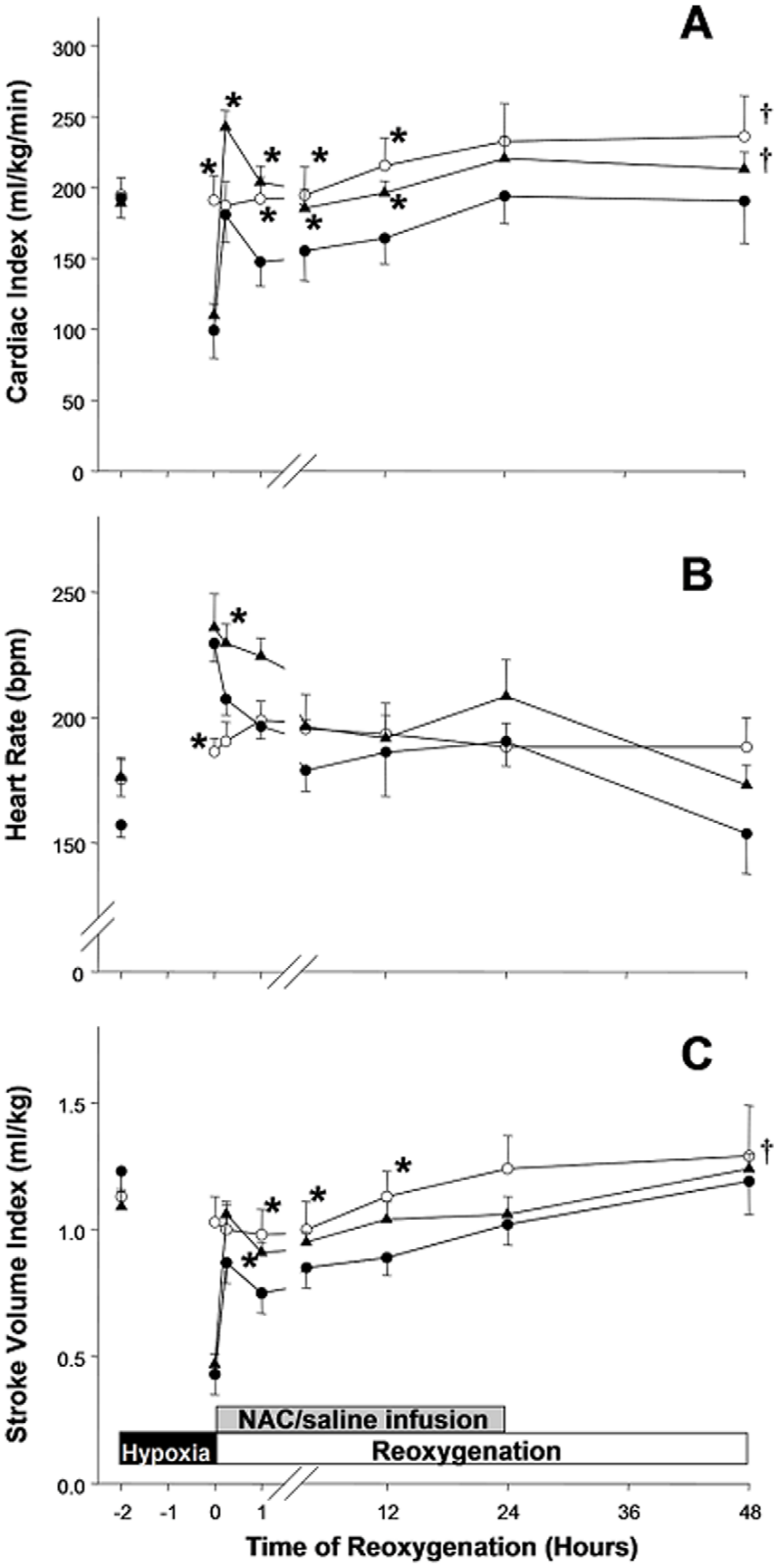
Effects of NAC on cardiac performance. Temporal changes in (A) cardiac index, (B) heart rate and (C) stroke volume index in sham-operated piglets (○, without hypoxia and reoxygenation, n = 6), hypoxia piglets receiving either saline (•, H−R control, n = 8) or NAC (▴, n = 8) at 5 min after reoxygenation. *p<0.05 vs. H−R control group at corresponding time point, ^†^p<0.05 vs. H−R controls during reoxygenation period (2-way repeated measures ANOVA).

Of H−R control piglets, CI decreased significantly at the end of hypoxia and rebound immediately upon resuscitation (Fig. 2A). Thereafter, CI gradually deteriorated and was lower than the baseline value throughout the first 12 h of reoxygenation period (p<0.05). NAC-treated piglets had CI higher than the baseline value initially, which was maintained at the baseline value throughout the reoxygenation period. Overall, CI of NAC-treated piglets was significantly higher than that of H−R control piglets throughout the reoxygenation period (Fig. 2A).

Heart rate increased significantly in hypoxic piglets (Fig. 2B). At 1 h of reoxygenation, heart rate of NAC-treated group was higher than that of both H−R control and sham-operated groups (224±7 vs. 198±8 and 191±5 beats/min, respectively; p<0.05). However, there were no statistical differences among all experimental groups thereafter (Fig. 2B).

The pattern of changes in stroke volume index corresponded to that observed with CI (Fig. 2C). The stroke volume index of H−R control piglets was significantly lower than that of sham-operated groups during the reoxygenation period.

**Figure 3:**
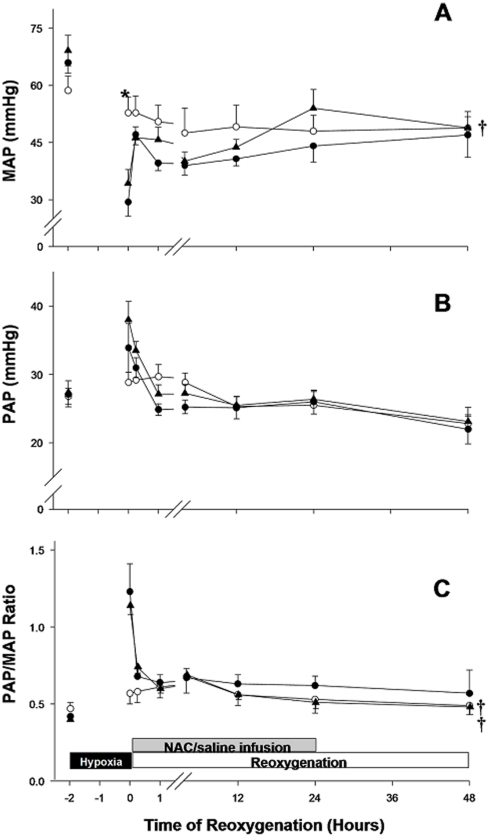
Effects of NAC on blood pressure. Temporal changes in (A) mean arterial pressure (MAP), (B) pulmonary artery pressure (PAP) and (C) PAP/MAP ratio in sham-operated piglets (○, without hypoxia and reoxygenation, n = 6), hypoxia piglets receiving either saline (•, H−R control, n = 8) or NAC (▴, n = 8) at 5 min after reoxygenation. *p<0.05 vs. H−R control group at corresponding time point, ^†^p<0.05 vs. H−R controls during reoxygenation period (2-way repeated measures ANOVA).

MAP decreased to 45% of the normoxic baseline value after 2 h of hypoxia (p<0.05)(Fig. 3A). The MAP of H−R controls rose immediately after reoxygenation but then gradually fell and remained lower than the baseline value throughout the recovery (p<0.05)(Fig. 3A). The MAP of NAC-treated piglets increased 12 h after reoxygenation and was the about the same level as the sham-operated group afterward (Fig. 3A). The PAP of hypoxic piglets was significantly higher than the normoxic baseline value at 2 h of hypoxia and remained elevated during the first 15 min of reoxygenation (Fig. 3B). The PAP then normalized at 1 h of reoxygenation with no difference among groups (Fig. 3B). The PAP/MAP ratio of H−R controls was higher than that of sham-operated and NAC-treated groups throughout the reoxygenation period (p<0.05)(Fig. 3C).

### Effects of NAC treatment on systemic oxygen transport

**Figure 4:**
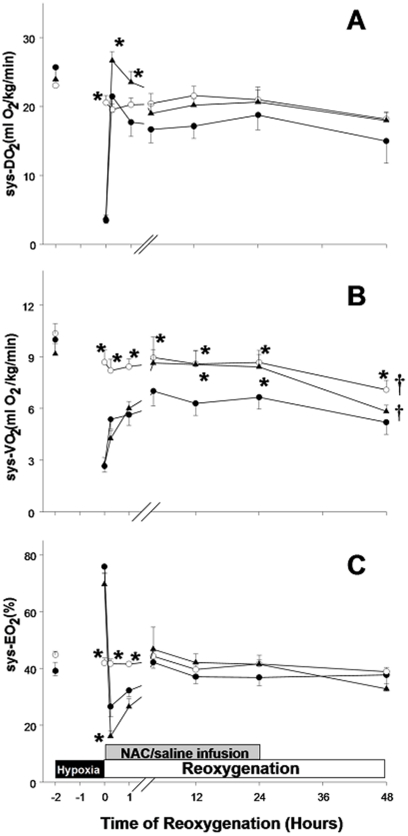
Effects of NAC on systemic oxygen delivery and extraction. Temporal changes in (A) systemic oxygen delivery (sys-DO_2_), (B) systemic oxygen consumption (sys-VO_2_) and (C) systemic oxygen extraction (sys-EO_2_) in sham-operated piglets (○, without hypoxia and reoxygenation, n = 6), hypoxia piglets receiving either saline (•, H−R control, n = 8) or NAC (▴, n = 8) at 5 min after reoxygenation. *p<0.05 vs. H−R control group at corresponding time point, ^†^p<0.05 vs. H−R controls during reoxygenation period (2-way repeated measures ANOVA).

Systemic oxygen delivery was significantly decreased in hypoxic piglets at 2 h of hypoxia, and rebounded to the level of sham-operated after reoxygenation with 100% O_2_ (Fig. 4A). The oxygen delivery of NAC-treated animals was higher than that of H−R controls (p<0.05), and returned to a level similar to that of the sham-operated group after switching to room-air. The oxygen delivery of H−R control group was low throughout the recovery, but was not significantly different from the other two groups. Systemic oxygen consumption of H−R controls increased gradually after reoxygenation but remained significantly lower than baseline value throughout the reoxygenation period (p<0.05)(Fig. 4B). NAC treatment improved systemic oxygen consumption to baseline value at 4 h after reoxygenation, and maintained the same level as sham-operated group throughout the observation period (Fig. 4B). The systemic oxygen consumption of NAC-treated piglets was significantly higher than that of H−R controls. Corresponding to the better oxygen delivery after reoxygenated with 100% O_2_ in NAC-treated animals, the systemic oxygen extraction was lower than that of H−R controls. The systemic oxygen extraction returned to the level of sham-operated in both H−R groups after reoxygenated with room-air and maintained at a level similar to that of sham-operated piglets thereafter (Fig. 4C).

### Effects of NAC on various biochemical and oxidative stress markers in myocardial tissues

Following H−R, the myocardial contents of total glutathione, GSSG and glutathione redox ratio (GSSG:GSH) on left ventricle of the H−R controls was not different from those of the sham-operated group (Table 2). Myocardial total glutathione, but not GSSG and redox ratio, was increased in NAC-treated animals (Table 2).

**Table 2:** Effects of NAC on myocardial contents of glutathione, lipid hydroperoxides (LPO), caspase-3 and lactate levels after hypoxic-reoxygenation.

Tissue content	Sham-operated	H-R Control	NAC	p value (ANOVA)
**Glutathione (nmol/mg protein)**				
GSH	399.6±36.8	352.0±16.5	441.5±8.4*	0.047
GSSG	8.87±1.3	8.16±1.6	13.7±1.9	0.054
GSSG/GSH	0.02±0.003	0.02±0.004	0.03±0.004	0.372
**LPO (nmol/mg protein)**	9.42±2.4*	28.1±4.8	16.7±2.7*	0.007
**Caspase-3 (Unit/mg protein)**	0.31±0.1*	0.71±0.1	0.42±0.1*	0.002
**Lactate (µmol/mg protein)**	2.71±0.2*	5.92±0.4	2.96±0.1*	0.016

*p<0.05 vs. H−R controls.

The myocardial LPO accumulation of H−R control piglets was significantly higher than those of sham-operated piglets, and the LPO accumulation was significantly reduced by NAC treatment (Table 2). Similarly, the increased activity of myocardial caspase-3 of H−R controls (vs. sham-operated piglets, p<0.05) was significantly reduced by NAC treatment (Table 2). The myocardial caspase-3 activities correlated positively with LPO accumulation (r = 0.53, p<0.05).

The myocardial lactate content of control piglets was higher than that of sham-operated group (p<0.05), whereas NAC significantly reduced the myocardial lactate content (Table 2). Interestingly, the lactate content was positively correlated with LPO accumulation and caspase-3 activity (r = 0.42, p<0.05 and r = 0.67, p<0.001; respectively). Furthermore, the change in CI after H−R (area under the curve) was negatively correlated with myocardial LPO, caspase-3 and lactate (r = −0.47, −0.56 and −0.47; all p<0.05, respectively).

### Effect of NAC on plasma cardiac troponin-I and lactate production

**Figure 5:**
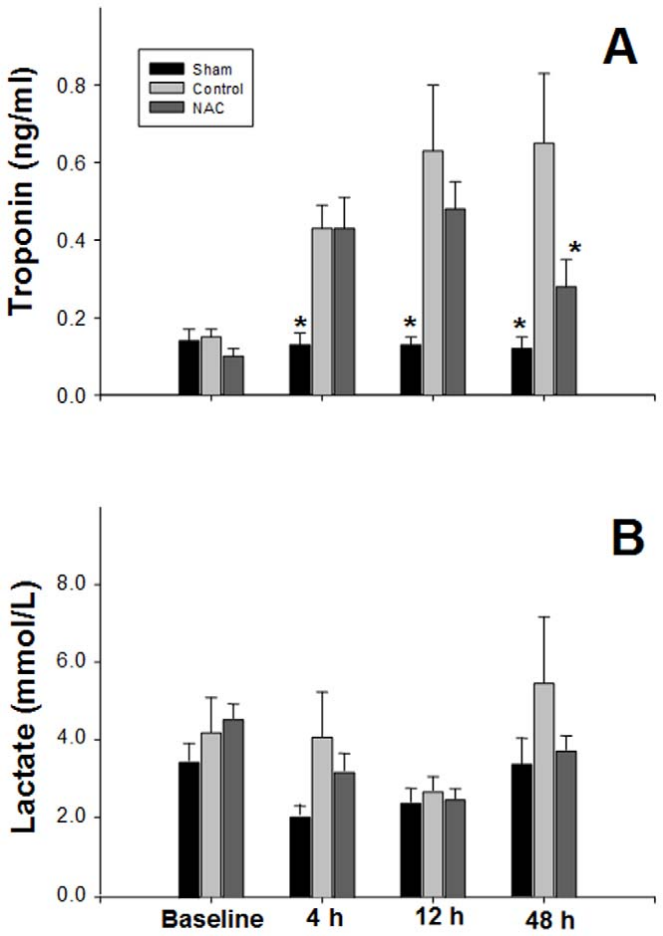
Effects of NAC on plasma troponin I and lactate. Changes in plasma (A) cardiac troponin I and (B) lactate concentrations in sham-operated (n = 6), H−R control (n = 8) and NAC-treated (n = 8) piglets at 4 h, 12 h, and 48 h after reoxygenation. *p<0.05 vs. H−R control group at corresponding time point.

The plasma cTnI concentration of sham-operated piglets did not change during the experimental period (Fig. 5A). Plasma cTnI of H−R controls increased gradually and was significantly higher than the sham-operated group throughout the reoxygenation period (Fig. 5A). As compared with H−R controls, animals treated with NAC gradually decreased the cTnI concentration and was significantly lowered at the end of the experiment (Fig. 5A).

Although plasma lactate concentrations of the H−R control group were slightly higher than both sham-operated and NAC-treated groups throughout the experimental period, the difference was not significant (Fig. 5B).

## Discussion

We hereby observed that post-resuscitation NAC infusion significantly improved the overall cardiac performance, particularly at the early phase of reoxygenation, consequently normalized the systemic oxygen delivery of H−R newborn piglets throughout reoxygenation. NAC treatments also attenuated myocardial LPO accumulation, caspase-3 activity as well as lactate content, and reduced the plasma cTnI concentration by the end of reoxygenation period.

The cardiac dysfunction after resuscitation contributes to the mortality and morbidity of neonates with perinatal asphyxia [21], [23]. Depending on the diagnostic criteria, cardiac dysfunction was observed in 29−67% of asphyxiated neonates [1], [2], [3]. Cardiac function can remain diminished with poor cardiac output and hypotension in the first 24−48 h after birth [27], similar to what we observed in our model of neonatal asphyxia. Using state-of-the-art technique such as functional echocardiography to determine cardiac function will be extremely revealing and useful. Nonetheless, as the result of poor systemic perfusion or delayed recovery from asphyxia, about 60% these cases were associated with adverse neurological outcome [2]. Thus, maintaining cardiac function after resuscitation may help to minimize further injury to asphyxiated neonates. As indicated by the lower CI and overall oxygen delivery, cardiac dysfunction did occur in H−R controls during reoxygenation.

In contrast to the cardiac dysfunction observed in H−R controls, treating the piglets with NAC significantly improved both the CI and overall oxygen delivery during the 48 h recovery. Plasma cTnI is a sensitive and specific marker of myocardial injury used for the diagnosis and prediction of the myocardial impairment [3], [23]. Similarly to those reported in adult patients with myocardial ischemia [28], [29], the plasma cTnI level of H−R controls peaked at 12 h after hypoxia and maintained high during the reoxygenation period. NAC treatment significantly decreased H−R-induced elevated cTnI concentration at the end of experimental period, indicating attenuated myocardial injury. Of note, this was associated with the significant reduction in myocardial lactate content, which was negatively correlated with CI. Interestingly, Harrison et al suggested that the increase in oxygen delivery could account for the beneficial effect of NAC in patients with fulminant hepatic failure [30]. Taken together, our results demonstrated that NAC could elicit a prolonged improvement in cardiac recovery in newborn asphyxiated piglets.

NAC is a precursor of L-cysteine and reduced glutathione [15]. It releases thione and converts glutathione into reduced form of GSH which is exhausted during hypoxia and ischemia [16]. Similarly to that reported previously in our acute study [9], a significant increase in myocardial GSH, but not GSSG and redox ratio, was observed in piglets receiving NAC treatment. Although the myocardial contents and redox ratio of glutathione were similar in H−R control and sham-operated group at the end of the experiment (48 h), the apparent contradiction could be due the fact that the endogenous glutathione system may have been restored during the prolonged recovery period. Furthermore, in addition to direct conversion from NAC, the increase in GSH in NAC-treated piglets may also be due to increased GSSH-reductase activity. Interestingly, oxidative stress has been shown to stimulate pentose-phosphate pathway that generates NADPH, a necessary cofactor for GSSH-reductase to maintain cellular GSH [31], [32]. As it has been shown previously that myocardial injury can be minimized by enhancing the glutathione content [33], [34], we speculate that replenishing endogenous GSH may, at least in part, account for the beneficial effect of NAC in improving cardiac recovery.

Associated with the impaired cardiac function, increases in myocardial LPO and caspase-3 were observed in H−R controls. Interestingly, the negative correlation between CI with both LPO accumulation and caspase-3 activity in the left ventricle may reflect the involvement of ROS in its pathogenesis. Indeed, the correlation between oxygen concentrations used in neonatal resuscitation and myocardial injury has been demonstrated [35]. ROS formed during oxidative stress can initiate lipid peroxidation, oxidize proteins and cause apoptosis cascades, all potentially damaging to normal cellular function. Complementary to our findings, reduced cardiac function has been observed in hearts perfused with various ROS generating systems [36], [37]. Therefore, these findings indicate that cardiac dysfunction in hypoxic newborn piglets observed after reoxygenation is associated with ROS-induced oxidative stress. Treating H−R piglets with NAC significantly attenuated the increased accumulation of myocardial LPO. This observation is consistent with the previous report showing that NAC can reduce ROS generation by its direct scavenging action [19], [38]. Interestingly, NAC has also been reported to attenuate the I−R-induced increase in peroxynitrite, a potent oxidant for tissue protein and lipid oxidation and detrimental effects on myocardial function [39], [40].

Similarly to previous reports of H−R or I−R [41], [42], increased caspase-3 activities were observed in the left ventricular myocardial tissue obtained from our H−R piglets. Caspases, particularly caspase-3, are involved in the apoptotic process [43], [44]. By using caspase inhibitor at different stages, it has been shown that only those caspases activated during reoxygenation were responsible for H−R induced apoptosis [45]. Reducing myocardial caspase-3 activity by various pharmacological therapies has been shown to minimize myocardial infarct size as well as myocardial injury [41], [42]. Previous studies have demonstrated that NAC could prevent apoptotic death of neuronal cell *in vitro* [46], [47]. We found that post-resuscitation treatment with NAC significantly reduced the activated caspase-3 levels after H−R. Interestingly, it is documented that cytochrome C released from mitochondria under oxidative stress can participate in the formation of apoptosome, the caspase-activating complex [48]. Regardless of the underlying mechanism, we demonstrated that the change in CI after H−R was negatively correlated with myocardial caspase-3 activity. Thus, our results suggest that caspase-3 may play an essential role in the worsening of cardiac function after H−R. NAC treatment may attenuate the apoptotic process through a reduction of ROS accumulation. This speculation is further supported by the positive correlation between myocardial activated caspase-3 and LPO.

Of note, NAC has been attempted without significant benefit in neonates at risk for bronchopulmonary dysplasia [49]. While the organ selectivity of NAC-induced protection remains to be investigated, in this setting of acute injury of H−R, we speculate the prolonged protective effects of NAC may be related to its anti-oxidative and anti-apoptotic effects in preventing myocardial injury. There were some limitations in this study. Firstly, asphyxiated neonates commonly have reduction of cardiac output [17], or stroke volume [50] within 24 h after birth, and require inotropic support. In this model, although CI and stroke volume of hypoxic piglets decreased to approximately 50% of the baseline at 2 h of hypoxia with acidemia similar to that of asphyxiated neonates in the delivery room (pH<7.1, HCO_3_<12 mmol/L), this does not replicate the exact situation in clinical scenario. Secondly, in order to minimize the effects of CO_2_ on the hemodynamic changes and cardiac function, normocapnia of the animals in this study was maintained during the experiment, while asphyxiated neonates usually have hypercapnia and ventilation is required. Thirdly, mechanisms of the relationship between NAC-improved cardiac function and the reduction of elevated levels of tissue lactate, LPO and caspase-3 in the myocardial tissue of left ventricle need to be further investigated. Furthermore, although the dosage of NAC was based on our previous studies of acute experimentation and has also been shown to be safe for neonates, the information of L-cysteine levels and dose-response of NAC treatment will be important in the translation of our findings to clinical setting. Despite reduced mortality has been seen in the resuscitation of asphyxiated term neonates with room air, compared to those with 100% oxygen, neonatal resuscitation with 100% oxygen remains a common practice in many centers, especially in community hospitals before the arrival of transport team. Recently, the guideline on the use of supplementary oxygen during neonatal resuscitation has been revised and it is recommended to start with 21% oxygen [51]. The cardiac protective effects of NAC when 21% oxygen is used during resuscitation will be interesting. Finally, it is interesting to further examine the role of anti-oxidants during neonatal resuscitation while we studied a state of excessive oxidative stress and its related injury, which is not uncommon in clinical conditions including uncontrolled hyperoxic resuscitation of asphyxiated neonates, cardio-pulmonary bypass and veno-arterial extracorporeal membrane oxygenation.

## Conclusions

In summary, post-resuscitation administration of NAC results in prolonged improvement in cardiac function and systemic oxygen delivery in asphyxiated newborn piglets 48 hours after H-R insult. Although our results demonstrate that NAC can also reduce LPO accumulation and caspase-3 activity, the exact mechanism on reducing oxidative stress still remains to be elucidated.
